# The association of dairy cattle longevity with farm level technical inefficiency

**DOI:** 10.3389/fvets.2022.1001015

**Published:** 2022-10-14

**Authors:** Ruozhu Han, Monique Mourits, Henk Hogeveen

**Affiliations:** Business Economics Group, Department of Social Sciences, Wageningen University, Wageningen, Netherlands

**Keywords:** dairy, culling age, lifetime milk production, input-specific technical efficiency, data envelopment analysis (DEA)

## Abstract

Prolonging dairy cattle longevity is regarded as one of the options to contribute to a more sustainable milk production. Cattle longevity is a direct result from culling decisions, which is primarily driven by economic considerations. As a consequence, at the herd level, cattle longevity can have effects on the efficiency of dairy production. This study investigates the technical inefficiency of dairy input, and its association with cattle longevity under Dutch commercial dairy production conditions, using a two-stage data envelopment analysis (DEA) approach. First, the technical inefficiency of capital, labor, land, seed & crop protection expenses, veterinary services, livestock purchase & services, feed purchase, miscellanea, livestock units and total input on total farm revenues was computed using DEA. Secondly, a bootstrap truncated regression analysis was applied to identify the association of cattle longevity with the evaluated input-specific and total input scores for technical inefficiency. Data were compiled from performance and accountancy records of 1,037 commercial Dutch dairy herds over the period of 2007 to 2014. In general, Dutch dairy farms displayed a relatively good overall technical efficiency, represented by an average inefficiency score of 0.09. The economic benefit of extending cattle longevity was evidenced by the negative association of cattle longevity with total input inefficiency. Of the evaluated inputs, the utilization of livestock units and feed was most efficient, with inefficiency scores below 0.26. This contrasts with the poor input efficiency of capital and livestock purchase & services with inefficiency scores around 0.52. Although the strength of the evaluated associations was generally low, the regression results illustrated that, except for labor, the age of culled cows was significantly negatively associated (*P* < 0.05) with each of the input inefficiencies. This contrasts with the significant associations of input inefficiencies with lifetime milk production, which were mostly positive. Since lifetime milk production is driven by length of cattle lifespan in combination with production level of the cows, the reverse direction of the associations with the two longevity indices illustrates that prolonging cattle longevity can improve efficiency performance of a dairy farm as long as the milk yield per cow remains unchanged.

## Introduction

Longevity of a dairy herd is reflected by the average age at which cows in the herd are culled (1, 2). In the Netherlands, the average age of culled dairy cows is 5.9 years (3, 4). This average age of culling is far below the potential natural lifespan of dairy cows (5). Therefore, increased longevity is perceived by society as a relevant indicator of animal welfare (6). Moreover, prolonging dairy cattle longevity is one of the potential options to contribute to a more sustainable milk production (1, 2), by reducing GHG emissions from youngstock rearing (7).

Within a commercial dairy herd, cattle longevity directly results from culling decision, which are primarily driven by economic considerations, by comparing the expected performance of present cows with the expected future performance of the available replacement cows. In the last two decades, technical efficiency has been widely used to measure the economic performance of dairy farms [e.g., (8, 9)]. Unlike accounting analysis (10), technical efficiency analysis is able to consider monetary as well as non-monetary inputs and outputs such as herd and land size and minimizes the impact of price volatility on farm's inputs and outputs (11). A farm is technical efficient if it produces a maximum output (total farm revenues) with a minimum amount of inputs, such as labor, feed, and equipment. Dairy farmers have greater autonomy to adjust the expenses on inputs rather than on output. Therefore, it is crucial for a dairy farm to promote the efficiency of inputs expenses. One of the options to adjust expenses on inputs is to prolong the longevity of dairy cattle, because that reduces the need for young stock and, therefore, reduces the need for inputs. To date, only a limited number of studies have been conducted on the association of prolonged cattle longevity on farm efficiency [e.g., (12, 13)]. More specifically, insights on the association between longevity and the efficiency of input specific use (e.g., the use of feed and labor) are lacking. The association of a prolonged cattle longevity with a farm's technical efficiency is expected to vary with the type of input resource used. As increased longevity could reduce costs associated with the rearing of replacement heifers and increase average herd milk production due to an increase in higher producing age groups (10, 14), but at the same time could also result in increased health and reproduction problems (15). In the total input technical score of a dairy farm, these potential opposing impacts might cancel each other out. These trade-offs between positive and negative impacts of longevity on farm efficiency make it difficult to advise farmers in their cattle longevity management. Insight in the association between dairy farm longevity and input-specific technical efficiency is, therefore, useful.

The objectives of this study was therefore (i) to measure the total input and input-specific technical inefficiency of dairy farms and (ii) to explore the association of cattle longevity with technical inefficiency under Dutch production conditions.

## Materials and methods

To achieve the indicated objectives, data envelopment analysis (DEA) was employed to measure technical inefficiency scores of specified inputs used to produce milk, followed by a bootstrap truncated regression model to identify the association of cattle longevity with input-specific technical inefficiencies. Data for this study were compiled from annual performance and accountancy records on Dutch commercial dairy herds during the period of 2007–2014.

## Methodology

### Input-specific DEA model

DEA is a non-parametric method to estimate the relative efficiency of decision-making units (DMUs; in this study farms). Unlike parametric methods, DEA is able to estimate efficiency with minimal prior assumptions about the production technology by which inputs are converted to an output. The production technology is, represented by the input requirement set of each DMU by comparing the level of inputs and outputs (16, 17). The best levels of inputs and outputs among those DMUs are located on the so-called efficient frontier. In this study, we consider *i* (*i*=1,…,N) farms (or DMUs), employing a number of variable and quasi-fixed inputs (X)^1^ to produce a single output Y. Under the assumption that returns to scale are variable (VRS),^2^ the production technology T is characterized by the input requirement set:


(1) $$\begin{array}{l} T\left(y\right)\;=\;\left\{\left(x,y\right)\right\} \end{array}$$

or non-parametrically as


(2) $$\begin{array}{l} T(y)\;=\;\{(x:Y^{\prime }\lambda \geq y_{i},\;X^{\prime }\lambda \leq x_{i},L^{\prime }\lambda =1,\;\lambda \geq 0)\} \end{array}$$

with all quantities being non-negative. Y denotes the (N^*^1) vector of output and *y*_*i*_ the output level of farm *i*. X denotes the matrix of inputs and *x*_*i*_ the vector of inputs for farm *i*. λ denotes the vector of farm-weights which indicates the observations to which a given farm is compared to. L denotes the vector of farms.

Following the definition of the production technology, a directional distance function was applied to determine the potential reduction of the amount of a specific input to achieve the same amount of output and using the same quantity of other inputs (18, 19). The directional distance function, representing the inefficiency scores, measures the amount that a given individual farm observation can be projected in the direction *g*_*x*_ until it reaches the efficiency frontier (20). Within this function, *g*_*x*_ denotes the directional vector associated with each input X. This input oriented method satisfies the condition that a farmer has limited capacity to increase the amount of milk production under a milk quota regimen, as was the case in the Netherlands during the reflected period of 2007–2014. To simplify the interpretation, inefficiency scores (θ) were calculated by a relative comparison of the actual performance score with the efficiency performance score using the following directional distance function:


(3) $$\begin{array}{l} \vec{D}\left(x,y;\;g_{x}\right)=\;\max\left\{\theta \;|\left(x-\theta g_{x}\right)\;\epsilon \;T\left(y\right)\right\} \end{array}$$

The inefficiency term (θ) is a vector for each farm concerning the separate inputs, respectively. Under the condition of VRS, this linear programming problem can be formulated as follows:


(4) $$\begin{array}{l} \vec{D}\left(x,y;g_{x}|VRS\right)=max\;\theta \end{array}$$

Subject to


(5) $$\begin{array}{l} \overset{N}{\underset{i=1}{\sum }}\lambda_{i}Y\;\geq \;y_{i} \end{array}$$

(6) $$\begin{array}{l} \overset{N}{\underset{i=1}{\sum }}\lambda_{i}X\;\leq \;x_{i}-\;\theta g_{x} \end{array}$$

(7) $$\begin{array}{l} \overset{N}{\underset{i=1}{\sum }}\lambda_{i}\;=1 \end{array}$$

(8) $$\begin{array}{l} \lambda_{i}\;\geq 0 \end{array}$$

The inefficiency scores (θ) range from 0 to 1, where a value of 0 represents a fully efficient farmer, located on the efficient frontier. In order to capture the different farm conditions across time with our multi-dimensional data, the linear programming calculations were carried out for each year separately to account for annual differences in the production conditions. Consequently, the inefficiency scores (θ) were obtained for each input and each year.

### Truncated bootstrap regression

To associate longevity with the inefficiency scores (θ), bootstrap truncated regression modeling was applied by which farm characteristics were regressed onto farm inefficiency scores. This regression modeling was computed separately for total input and each input-specific inefficiency score. The formal model looks as follows:


(9) $$\begin{array}{l} \theta \;=\;\alpha I\;+\;\gamma T\;+\;\beta Z\;+\varepsilon \end{array}$$

Where θ is the vector of inefficiency scores across all years of farmers with θ > 0 obtained for each input derived from the input-specific DEA model. *I* is a vector of ones with length N, and α denotes the parameter for the intercept. *T* denotes the year-dummies that were included to correct for the differences in the inefficiency frontier between the different years and γ denotes the corresponding vector of parameters. *Z* denotes the matrix of farm characteristics (Table 1), among which we find cows' longevity, and β denotes the vector of parameters for these data. ε denotes the error term.

**Table 1:** Descriptive statistics of the selected covariates in the bootstrap truncated regression based on the herd data from 2007 to 2014 (*n* = 7,782).

**Variables**		**Mean**	**SD**	**Min**	**Max**
Age of culled cows (year)		5.9	0.8	4.7	7.4
Lifetime milk production (ton)		31.5	7.7	20.2	45.0
Production intensity (ton/ha)		15.2	4.2	9.6	22.3
Herd expansion ratio		1.1	0.2	1.0	1.4
Heifer ratio		0.23	0.06	0.13	0.33
		***N*** **obs**			
Successor	No	4,880			
	Yes	2,902			
Soil type	Sandy soil	5,439			
	Other soil	2,343			

Since the input-specific inefficiency score for a farm was defined relative to the frontier representing the best practice, estimated DEA inefficiency scores are serially correlated. As this violates the basic assumption of independence within sample values, the direct use of the estimated scores in a regression analysis to evaluate differences in efficiency among farmers in relation to longevity could result in invalid interpretations. In order to overcome this difficulty, a single truncated bootstrap regression (21) was applied. Estimated inefficiency scores θ derived from the input-specific DEA model were used to reckon β and estimate σ_ε_ by the method of maximum likelihood in truncated regression. In order to obtain a set of bootstrap estimates (β^*^, ${\sigma_{\varepsilon }}^{*}$), the next three steps were looped over 2,000 times. Firstly, the error term ε_*i*_ was assumed to be an N (0, ${\sigma_{\varepsilon }}^{2}$) distribution with left-truncation at (0- βZ). Secondly, for each *i* = 1,…,N, $\theta_{i}^{*}$ was estimated by $\theta_{i}^{*}=\;\alpha I_{i}\;+\;\gamma T_{i}\;+\beta Z_{i}\;+\varepsilon_{i}$. Lastly, the estimated (β^*^, ${\sigma_{\varepsilon }}^{*}$) was obtained by estimating the truncated regression of $\theta_{i}^{*}$ on *Z*_*i*_. In each iteration, truncated regression model above is computed. Consequently 2,000 coefficient estimates were obtained. The mean of these estimates was used as the final coefficient estimate and the distribution of them was used to conclude on the statistical significance at different confidence levels.

### Available data

Annual farm accountancy data provided by a Dutch accounting agency (Flynth, Arnhem, the Netherlands) was merged with herd characteristics data derived from the Cattle Improvement Cooperative (CRV, Arnhem, the Netherlands) with consent of their associated farmers. Data was anonymized so that we could not trace it back to individual farmers. A contract between the data providers and the university guaranteed proper data management procedures. The resulting dataset consisted of comprehensive data with information on 2,362 herds over the period 2007–2014. The economic performance of the herds was indicated by accountancy data on total revenues, quasi-fixed costs and variable costs. The data on herd characteristics covered information on cattle longevity, production intensity, herd size, and general farm characteristics (e.g., heifer ratio).

### Data editing

To ensure that the analysis was representative of commercial dairy milk production circumstances, farms included in the analysis needed to adhere to the following five conditions: continuous farming throughout all evaluated years, >75% of the total revenue stems from milk sales, no by-product revenue from milk processing (e.g., farmhouse cheese production), no organic farming, and a dairy herd size ≥30 cows. After enforcing these conditions, 7,782 herd-year observations from 1,036 herds with complete information were kept for further analysis.

One output and nine input sources were defined for the technical efficiency analyses. Output (Y) was reflected by the indicated total farm revenue (€), which was deflated by a Tornqvist index based on the reference year of 2010. Total farm revenue was an aggregate of milk revenue, meat revenue and revenue generated from feed sales. The selected inputs *X*_*i*_ (*i* = 1, ….9) included (i) capital as reflected by the balance sheet values collected in a consistent manner by the accounting firm (Flynth) for farm buildings and machinery, (ii) labor including family and hired labor as indicated by the number of full-time employees (**FTE**), (iii) land as measured by the area used for production, (iv) seed & crop protection expenses, indicating expenses of seed and crop protection and fertilizer; (v) veterinary services, containing the expenses for veterinary services, artificial insemination, breeding and control, AI breeding & milk production recording and embryo transplantation; (vi) livestock purchase & services, containing expenses for young cattle rearing carried out by third parties, livestock purchases, and expense of work by third parties. As most of Dutch dairy farm rear their own youngstock, the majority of the costs within this category is from work by third parties; (vii) feed, reflecting expenses of all feed purchases, being mainly concentrates purchase; (viii) miscellanea comprising expenses for litter and other remaining variable costs, and lastly, (ix) livestock units containing information on the number of cattle kept. Livestock units were calculated based on the livestock reference units as applied by EUROSTAT.^3^ All monetary expenses were expressed in Euro and were deflated using individual price indices obtained from EUROSTAT. The descriptive statistics of the selected inputs and output variables are displayed in Table 2.

**Table 2:** Descriptive statistics of the selected DEA variables based on the herd data (*n* = 7,782) from 2007 to 2014.

**Variables**	**Mean**	**SD**	**Percentile_5**	**Percentile_95**
**Inputs** Capital (€)	384,709	343,745	65,700	1,037,666
Labor (FTE)	1.88	0.73	1.00	3.00
Land (ha)	51	26	25	93
Seed & crop protection expenses (€)	12,038	6,654	4,640	23,844
Veterinary service (€)	20,575	9,760	9,056	38,507
Livestock purchase & services (€)	23,531	17,560	5,606	52,247
Feed (€)	66,550	42,611	23,418	142,976
Miscellanea (€)	10,249	5,966	3,312	21,709
Livestock units	127	67	60	231
**Output** Revenue (€)	302,193	167,868	132,661	573,205

Cattle longevity is the factor of interest in the second stage of the modeling. Two annually (over the production year) averaged indices were selected to measure cattle longevity: age of culled milking cows (year) (Z_1_) and lifetime milk production of culled cows (ton) (Z_2_). According to the definition used by Fetrow et al. (22), the number of culled cows represented milking cows after first calving that were removed from the dairy herd for slaughter, salvage or death within a production year. Animals sold for production purposes to other dairy farms were excluded from this number. Besides cattle longevity, five covariates were selected as explanatory variables based on an expected association with longevity and farm technical inefficiency. These covariates included production intensity (Z_3_), herd expansion (Z_4_), heifer ratio (Z_5_), and successor availability (yes/no) (Z_6_). Since Dutch farms producing on different soil types (especially clay vs. sand) differ in milk revenues and costs for purchasing feed (10), soil type (sand/others) (Z_7_) was also taking into consideration. Production intensity, herd expansion and heifer ratio were derived from the registered data. Production intensity indicated the annual average milk production in tons per hectare. Herd expansion reflected the ratio of herd size change relative to the reference year 2007. In addition, the heifer ratio was calculated by dividing the number of first calving heifers by the number of milking cows in a given production year. The descriptive statistics of the selected covariates are presented in Table 1.

## Results

### Total input and input-specific technical inefficiencies

**Figure 1:**
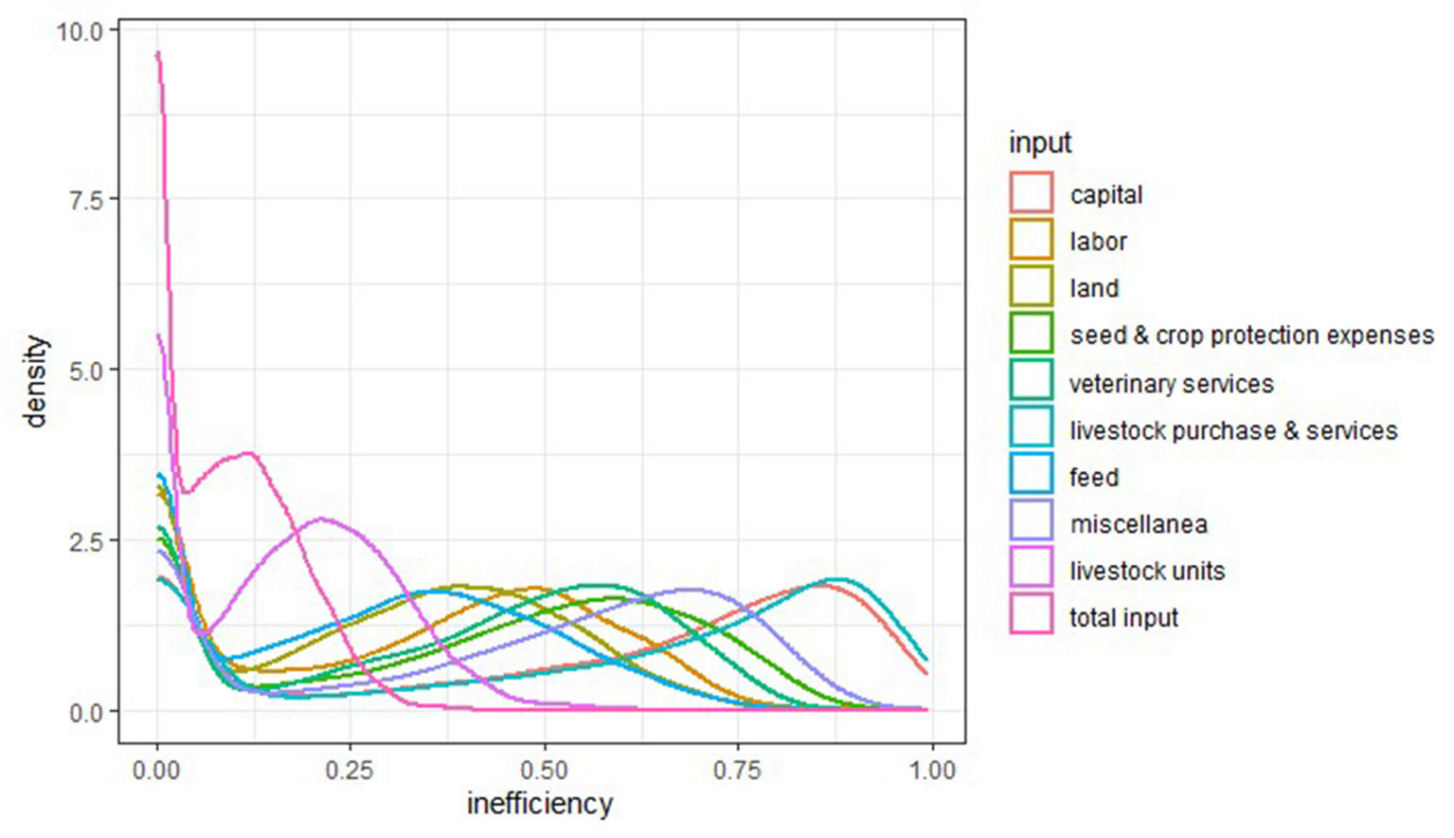
Density plot reflecting the inefficiency score distribution of the individual input factors and overall input over years.

The annual average inefficiency scores for each of the individual input sources as well as for total inputs are displayed in Table 3. The generic inefficiency performance of each input is arrayed by its corresponding mean value (Table 2) and distribution of inefficiency score across years (Figure 1). In general, total input is rather efficiently used as indicated by the average technical inefficiency over the evaluated period of 0.09 (where 0 denotes the best achievable efficiency). This means that, on average, farmers can decrease the use of overall inputs by only 9% to produce the same amount of output (total farm revenues). Inefficiency scores of the specific inputs are higher and vary substantially among each other. The utilization of livestock units and feed are the most efficient. Their mean inefficiency scores indicate that, on average, by employing the same quantity of other inputs, the same output can be achieved by decreasing the use of livestock by 16% or feed by 26%. On the other hand, the inputs of capital and livestock purchase & services can, on average, be reduced with 52 and 53%, respectively, to achieve the same amount of output, indicating a less efficient use of these inputs.

**Table 3:** Average annual inefficiency scores for capital, labor, land, seed & crop protection expenses, veterinary services, livestock purchase & services, feed, miscellanea, livestock units, and total inputs.

	**2007**	**2008**	**2009**	**2010**	**2011**	**2012**	**2013**	**2014**	**Mean^a^**
Capital	0.45	0.51	0.54	0.55	0.54	0.53	0.50	0.54	0.52
Labor	0.29	0.34	0.32	0.30	0.30	0.29	0.29	0.33	0.31
Land	0.24	0.28	0.34	0.29	0.26	0.28	0.28	0.27	0.28
Seed & crop protection expenses	0.36	0.41	0.46	0.41	0.35	0.36	0.35	0.38	0.39
Veterinary services	0.33	0.36	0.40	0.39	0.37	0.34	0.35	0.38	0.37
Livestock purchase & services	0.49	0.57	0.57	0.53	0.53	0.55	0.49	0.52	0.53
Feed	0.21	0.24	0.36	0.29	0.27	0.26	0.22	0.25	0.26
Miscellanea	0.39	0.47	0.45	0.45	0.42	0.43	0.38	0.42	0.43
Livestock units	0.14	0.17	0.17	0.16	0.18	0.17	0.15	0.17	0.16
Total input	0.07	0.09	0.10	0.09	0.09	0.09	0.08	0.09	0.09

a Mean value of each input-specific inefficiency score over evaluated period (2007–2014).

In order to present the distribution of input-based efficiency performance, a density plot was used to show the probability density function of each input inefficiency score over the evaluated period. Since input inefficiency scores within the population of evaluated farmers display a bimodal distribution, the degree of the bimodal spread of values is not observed when solely looking at the mean inefficiency score. The peaks of a density plot helps to display where the modes of the population are situated along the inefficiency interval (Figure 1). As a sizable number of farms lay on the efficient frontier for the individual inputs, all density curves show a peak at 0. However, the different input-specific inefficiencies vary considerably in the distribution for values larger than 0 (i.e., more inefficient farms). For example, the second mode for the inefficiency scores for livestock units lays around 0.24, whereas, this value for capital and livestock purchase & services lays around 0.87. This means that farmers are relatively comparable in their managerial performance per livestock unit, but that for other inputs, such as capital, the sample can be differentiated into farms that are very efficient and farms that are less efficient.

### Determinants of technical inefficiency

The results of the bootstrap truncated regression analysis are depicted in Tables 4, 5 corresponding to the models including culled cows age or lifetime milk production as longevity feature. In order to compare the goodness-of-fit of each models, the tables also provide Akaike information criterion (AIC).

**Table 4:** Results of bootstrap truncated regression models on technical inefficiency for, respectively, capital, labor, land, seed & crop protection expenses, veterinary services, livestock purchase & services, feed, miscellanea, livestock units, and total input with age of culled cows as longevity feature.

		**Capital**		**Labor**		**Land**		**Seed & crop protection expenses**		**Veterinary services**		**Livestock purchase & services**		**Feed**		**Miscellanea**		**Livestock units**		**Total input**	
Age culled cows (year)		−0.010	**	−0.004		−0.010	***	−0.009	***	−0.005	*	−0.010	***	−0.007	**	−0.014	***	−0.005	***	−0.005	***
Successor	No^a^																				
	Yes	0.014	**	0.105	***	0.026	***	0.020	***	0.013	***	0.043	***	0.011	***	0.013	**	0.006	**	0.015	***
Soil	Sand^a^																				
	Others	−0.002		−0.035	***	0.032	***	−0.030	***	−0.014	***	−0.017	***	−0.001		−0.008		−0.006	**	−0.004	**
Production intensity		−0.005	***	−0.003	***	−0.022	***	−0.012	***	−0.010	***	−0.010	***	−0.012	***	−0.012	***	−0.012	***	−0.009	***
Herd expansion		0.0002		0.035	**	0.010		−0.043	***	−0.006		0.057	***	−0.054	***	−0.029	*	0.044	***	0.015	**
Heifer ratio		0.121	**	0.037		0.049		0.148	***	0.118	***	0.116	**	0.095	**	0.107	**	0.001		0.026	
AIC		−1,237		−7,008		−4,303		−3,788		−4,589		−1,091		−6,168		−3,325		−11,458		−17,011	

^***^P < 0.01; ^**^P < 0.05; ^*^P < 0.10.

a This group was used as reference category in the regression analyzes.

**Table 5:** The results of bootstrap truncated regression models on technical inefficiency for, respectively, capital, labor, land, seed & crop protection expenses, veterinary services, livestock purchase & services, feed, miscellanea, livestock units, and total input with lifetime milk production as longevity feature.

		**Capital**		**Labor**		**Land**		**Seed & crop protection expenses**		**Veterinary services**		**Livestock purchase & services**		**Feed**		**Miscellanea**		**Livestock units**		**Total input**	
Lifetime prod (ton)		−0.0001		0.0011	***	0.0005	*	0.0004		0.0013	***	−0.0005		−0.0002		0.0003		−0.0027	***	−0.0006	***
Successor	No^a^																				
	Yes	0.014	**	0.105	***	0.026	***	0.020	***	0.012	***	0.043	***	0.011	***	0.013	**	0.007	***	0.015	***
Soil	Sand^a^																				
	Others	−0.003		−0.036	***	0.032	***	−0.031	***	−0.015	***	−0.017	***	−0.001		−0.009		−0.005		−0.004	*
Production intensity		−0.004	***	−0.004	***	−0.022	***	−0.012	***	−0.010	***	−0.010	***	−0.012	***	−0.012	***	−0.010	***	−0.009	***
Herd expansion		0.0002		0.038	**	0.012		−0.041	***	−0.002		0.055	***	−0.055	***	−0.028		0.035	***	0.013	*
Heifer ratio		0.154	***	0.069		0.091	***	0.184	***	0.156	***	0.139	***	0.113	***	0.160	***	−0.029		0.031	
AIC		−1,230		−6,994		−4,310		−3,782		−4,603		−1,086		−6,163		−3,306		−11,617		−17,015	

^***^P < 0.01; ^**^P < 0.05; ^*^P < 0.10.

a This group was used as reference category in the regression analyzes.

Except for the input labor, age of culled cows is significantly negatively (*P* < 0.05) associated with total input inefficiency and each of the input inefficiencies. However, the association is rather weak as indicated by the small values of the coefficients (ranging between −0.004 and −0.014). For example, the coefficient of age of culled cows on feed inefficiency is −0.007. It indicates that 1 year increase in the age of culled cows is associated with a decrease in the inefficiency of feed input by 0.007. Although the degree of association is very small, the results reveal that cattle longevity is associated with slightly better total input and input specific efficiency.

Similarly, the coefficient of the covariate production intensity displays significant (*P* < 0.01) negative relationships with each input inefficiency score, ranging from −0.003 to −0.022.

In addition, heifer ratio is positively associated (*P* < 0.1) with total input and most of the input specific inefficiencies, except for labor, land and livestock units. Herd expansion is only negatively associated with seed & crop protection expenses, miscellanea and feed inefficiency. Interestingly, for farms with a successor, the coefficients illustrate positive associations with total input and each of the input inefficiencies compared to farms without successor. In addition, farms with another type of soil other than sand undermine the utilization of agriculture area (positive association).

In the models regarding lifetime milk production as longevity index, lifetime milk production was significantly (*P* < 0.05) associated with the inefficiency of labor, land, veterinary service, livestock units, and total input. While the technical efficiency of total input was negatively associated with lifetime milk production, most of the significant input specific inefficiencies (labor, land, veterinary service) disclosed a positive relationships with lifetime milk production, except for the input livestock units. The association of other covariates (availability of successor, soil type, production intensity, herd expansion, heifer ratio) with each of the inputs inefficiency displayed similar results as in the model expressing longevity by the age of culled cow index.

## Discussion

Over the last decade, several studies have explored the overall technical efficiency performance of Dutch dairy farms [e.g., (13, 23)]. These studies indicated that Dutch dairy farms, in general, perform rather efficient in overall input usage which is similar as the inefficiency score of total input around 0.09 obtained from this study. However, inputs of a farm are comprised of several components, such as feed and health expenses, which are managed differently by farmers. Hence, the impact of prolonging cattle longevity can be different due to different inputs inefficiency. Since the overall technical efficiency performance of a dairy farm is not simply the summation of inputs specific efficiency performance, the utilization level of these individual inputs can hardly be derived from the overall technical efficiency performance, and vice versa. In this study, the combination of elaborate databases including the accountancy and longevity performance of commercial Dutch dairy farm provided a possibility to get insight into the technical efficiency of each input and determinants of input-based inefficiency.

Capital and livestock purchases & services had the highest technical inefficiency scores. In the density plots of inefficiency score distribution, a strong bimodal nature of those two inputs reveals that two different populations of farmers may underly the analysis. That means the farms are either very efficient or very inefficient in relation to capital and livestock purchases & services expenses. The covariates availability of a successor and heifer ratio could partially explain the inefficiency of capital and livestock purchase & services expenses given the regression results in the second stage of our research. Firstly, a farm with a potential successor aims to continue the business and/or may have a stronger educational background, which is more likely to result in innovative investments to better cope with future changes in the production condition (24). Therefore, innovative investments in buildings and equipment could explain the difference in input inefficiencies between farms with and without a successor. Additional data exploration also reveals that the average capital differs among these two groups. Secondly, introduction of heifers to replace the culled cows involve large costs (25), contributing around 20% to the overall expenditure on a dairy farm (26). Farmers only start to earn back these costs when the net revenues realized from the milk production by the replacement animal cover the costs accrued during the rearing period. Previous research highlights that the costs of rearing a replacement heifer are not recovered until their second lactation (27). Consequently, farms with a higher heifer ratio use most inputs less efficient, i.e. capital, veterinary service, livestock purchase & services and miscellanea expenses.

After capital and livestock purchases & services, the input variable miscellanea (expenditures for litter, electricity, water, etc.), had the highest inefficiency score. This variable, however, represents, only a small amount (7%) of the total variable expenses (Table 2). From an efficiency point of view, the use of these inputs can be significantly improved, although the absolute effect on net returns will be limited. In contrast, the cost of feed, on average, accounts for 50% of total variable expenses (Table 2) which displayed the highest efficiency performance after livestock units utilization.

When using a regression model to analyze observational data, such as a truncated bootstrap regression, causality may be implied while not supported by the underlying data generating process. Although the analysis is based on the hypothesis that longevity affects input inefficiency, input inefficiency itself could potentially affect longevity (higher input inefficiency could lead to earlier culling), suggesting the presence of reverse causality. Reverse causality could harm the quality of the results of the regression model, indicating a limitation of the approach used.

The main results from the second stage truncated bootstrap regression indicate the benefit of extending cattle longevity for the economic performance of a dairy farm, as shown by the negative associations of total inefficiency scores with the two independent longevity indices. With regard to the input specific associations with longevity, the average age at culling was negatively associated with all input-specific inefficiencies. Although coefficients of those associations are small, these results do illustrate the potential benefit of extending the age of culling in improving the farm input efficiency performance. In contrast, the associations of average lifetime milk production with input-specific inefficiencies were mostly positive. Average lifetime milk production only showed a negative association with the inefficient utilization of livestock units. Since lifetime milk production is driven by two elements, i.e., lifespan (culling age) and production level of an individual cow, the opposing associations of input-based inefficiency scores with age at culling and lifetime production indicate that increasing age at culling could improve farm efficiency performance as long as the milk yield at cow level remains unchanged. The positive associations of lifetime milk production with veterinary services and labor inefficiency could be related to higher risk of health disorders caused by higher milk yields per cow. At the cow level, numerous studies have proven a strong association of high milk yield with health and fertility disorders, resulting in increased risk of culling (28–30). Since the results of the regression model do not indicate a negative impact on veterinary service and labor efficiency performance by extending the average culled cow age, the negative impact of extending lifetime milk production on veterinary service and labor efficiency could be mainly due to the stronger association with the milk production level of individual cows.

The negative association of livestock units with both longevity indices illustrates that extending lifespan of a dairy cow or increasing lifetime milk production per cow can achieve better livestock units efficiency. Since the variable livestock units represents cows from two age groups, viz. youngstock (before 1st calving) and milking cows, the negative associations are mainly based on the milk production of the group of dairy cows. With an increased culling age, the relative contribution of the number of dairy cows to the total number of livestock units on a farm increases, leading to a higher total milk production on the farm. In the case of the extended lifetime production, the increase in livestock unit performance directly results from an increased milk production per cow.

Cattle longevity is often seen as one of the possibilities to reduce the environmental footprint of dairy production (1, 2). The results of this study show that it is possible for a farm to work with a longer longevity without negative economic consequences and even some potential positive economic consequences.

## Conclusion

Despite, the relatively good overall technical efficiency of Dutch dairy farms, the efficiency performance varied for different inputs. Capital and livestock purchase & services expenses displayed poor efficiency performance. Overall technical inefficiency was negatively associated with longevity illustrating the potential economic benefit of extending cattle longevity. For associations of longevity with each input-based inefficiency score, except for score of labor, age of culled cows was significantly negatively associated with each of the input inefficiencies. In contrast, the significant associations of input inefficiencies with lifetime milk production were mostly positive. Since lifetime milk production is driven by length of cattle lifespan and individual cow production intensity, this reverse direction of associations of inefficiency scores with two longevity indices indicated that extending the cow age at culling may lead to improved economic performance of dairy farms as long as the milk yield at cow level remains unchanged.

## Data availability statement

The data analyzed in this study is subject to the following licenses/restrictions: A separate, independent, company did anonymize the data so that data could not be traced back to individual farmers. A contract between the data provider and the university guarantees proper data management procedures. Requests to access these datasets should be directed to henk.hogeveen@wur.nl.

## Author contributions

RH: data analysis and drafting the manuscript. MM: drafting the manuscript and critical revision of the article. HH: critical revision of the article. All authors contributed to the article and approved the submitted version.

## Funding

RH and availability of the data are financially supported by the Sino-Dutch Dairy Development Center (SDDDC) and China Scholarship Council (CSC). Funds received for open access publication fees.

## Conflict of interest

The authors declare that the research was conducted in the absence of any commercial or financial relationships that could be construed as a potential conflict of interest.

## Publisher's note

All claims expressed in this article are solely those of the authors and do not necessarily represent those of their affiliated organizations, or those of the publisher, the editors and the reviewers. Any product that may be evaluated in this article, or claim that may be made by its manufacturer, is not guaranteed or endorsed by the publisher.

## Footnotes

^1^Under the assumption that farmers can adjust quasi-fixed inputs in the long run, the quasi-fixed inputs, in this study, are also regarded as variable inputs.

^2^VRS permits constant but also increasing and decreasing returns to scale at different scale sizes (31).

^3^Livestock units = number of milking cows + 0.7 ^*^ number of youngstock > 1 year old + 0.4 ^*^ number of youngstock ≤ 1 year old.
