# Supplementary material for: The association of dairy cattle longevity with farm level technical inefficiency
Source: Front Vet Sci. 2022 Oct 14;9:1001015. doi: 10.3389/fvets.2022.1001015 (PMC9614276; doi:10.3389/fvets.2022.1001015)
Supplement: Supplementary file 1 [file Table_1.pdf]

SUPPLEMENTARY

Table 1. The results of bootstrap truncated regression models on technical inefficiency for, respectively, capital, labor, land, seed & crop protection expenses, veterinary services, livestock purchase & services, feed, miscellanea, livestock units and total input with age of milking cows as longevity feature.

		capital	labor	land	seed & crop protection expenses	veterinary services	livestock purchase & services	feed	miscellanea	livestock units	Total input
age cows (year)		-0.014 **	-0.009 *	-0.012 ***	-0.016 ***	-0.012 **	-0.022 ***	-0.004	-0.024 ***	-0.005 *	-0.007 ***
successor	no[1]										
	yes	0.014 **	0.105 ***	0.026 ***	0.020 ***	0.012 ***	0.043 ***	0.011 ***	0.013 **	0.006 **	0.015 ***
soil	sand[1]										
	others	-0.002	-0.035 ***	0.033 ***	-0.030 ***	-0.013 ***	-0.016 ***	-0.001	-0.007	-0.006 **	-0.004 *
production intensity		-0.005 ***	-0.003 ***	-0.022 ***	-0.012 ***	-0.010 ***	-0.010 ***	-0.012 ***	-0.012 ***	-0.012 ***	-0.009 ***
herd expansion		-0.001	0.034 **	0.009	-0.044 ***	-0.007	0.055 ***	-0.054 ***	-0.031 *	0.044 ***	0.014 *
heifer ratio		0.114 **	0.022	0.049	0.129 ***	0.098 **	0.084	0.105 ***	0.084 *	0.003	0.022
AIC		-1234	-6998	-4304	-3789	-4592	-1095	-6163	-3323	-11452	-17007

***P < 0.01; **P < 0.05; *P < 0.10

[1] This group was used as reference category in the regression analyzes
